# The cruel and unusual phenomenology of solitary confinement

**DOI:** 10.3389/fpsyg.2014.00585

**Published:** 2014-06-12

**Authors:** Shaun Gallagher

**Affiliations:** ^1^Department of Philosophy, University of MemphisMemphis, TN, USA; ^2^School of Humanities, University of Hertfordshire HatfieldHertfordshire, UK; ^3^Faculty of Law, Humanities and the Arts, University of WollongongWollongong, NSW, Australia

**Keywords:** solitary confinement, cruelty, intersubjectivity, induced autism, self

## Abstract

What happens when subjects are deprived of intersubjective contact? This paper looks closely at the phenomenology and psychology of one example of that deprivation: solitary confinement. It also puts the phenomenology and psychology of solitary confinement to use in the legal context. Not only is there no consensus on whether solitary confinement is a “cruel and unusual punishment,” there is no consensus on the definition of the term “cruel” in the use of that legal phrase. I argue that we can find a moral consensus on the meaning of “cruelty” by looking specifically at the phenomenology and psychology of solitary confinement.

A number of legal declarations prohibit “cruel” punishments. The Eighth Amendment to the United States Constitution (1791), for example, declares: “cruel and unusual punishments [shall not be] inflicted.” From the beginning, however, the wording was thought “too indefinite,” or “to have no meaning in it^1^.” It is still difficult to find a clear definition of “cruel” in the legal domain. The intent of the present paper is in line with a recommendation made by Radin (1978, p. 992), that the courts “must search for a deeper moral consensus on the meaning of cruelty in order to determine whether a specific punishment comports with current standards of decency.” Rather than looking to legal history, “legislative enactments, referenda or opinion polls” (Ibid), however, I propose that we look to a combination of philosophical and scientific methods that include phenomenology, psychology, psychiatry, developmental psychology, and neuroscience, to explicate the specific experiences of those who undergo punishments, with a view to formulating a deeper moral consensus^2^

My focus in this paper is limited to the practice of solitary confinement. Solitary confinement may differ from one prison to the next in the precise details of how it is carried out. I assume, however, that the common element is some high degree of isolation – the reduction or complete elimination of intersubjective contact between the prisoner and others for a significant amount of time. Accordingly, I’ll begin with an outline of some classic phenomenological concepts related directly to the notion of intersubjectivity. I’ll then show how these concepts are reinforced by developmental studies. The question then becomes: what happens when subjects are deprived of intersubjective contact? Here I’ll appeal to the notion of induced autism, and then look closely at the effects of solitary confinement. In the final section I return to the question of what constitutes cruel punishment.

## BASIC CONCEPTS IN THE PHENOMENOLOGY OF INTERSUBJECTIVITY

Phenomenological philosophy, which can be traced to the work of Edmund Husserl at the beginning of the twentieth century, has recently been incorporated into scientific studies of cognition, including embodied and enactive approaches to social cognition (e.g., Gallagher, 2001, 2005, 2012; Ratcliffe, 2006; De Jaegher et al., 2010). Phenomenology, even in its classical form, emphasizes the constitutive nature of intersubjectivity. I’ll briefly discuss three concepts from classical phenomenology directly relevant to this idea: being-with, transcendental intersubjectivity and intercorporeity.

Heidegger (1962) provides an analysis of human existence in which being-with (*Mitsein*) or being-with-others is part of the very structure of human existence, shaping the way that we are in the world. According to this notion, the social dimension is not an external add-on or supplement to our existence. Being-with does not signify that we are in-the-world first, and then because of that we come to be with others. In other words, our social nature does not depend on empirically encountering others; it is rather an *a priori* structure – the fact that others are in the world only has significance because our existence is structured as being-with. If one happens to be alone, one still has the structure of being-with – and “only as being-with can [one] be alone” (1985, p. 238). Heidegger goes on to further emphasize that this particular way of being-with co-determines other aspects of our existence, including our relations with the world around us: “By reasons of this *with-like* [*mithaften*] being-in-the-world, the world is always the one that I share with Others. The world of [human existence] is a *with-world* [*Mitwelt*]” (1962, p. 155/118). One encounters others primarily through one’s various projects, and even in terms of what one perceives. One’s projects equally implicate other people – as co-workers, intended recipients and so on. So the world in which we find ourselves cannot be extricated from our relations with other people – it is permeated with social relations. Heidegger also makes the point that being-with shapes our own self-experience. One’s *own* existence is something that one experiences in the kinds of pragmatic projects that one shares with others.

In effect, one doesn’t come to have a social constitution by way of interacting with others; one is “hard-wired” to be other-oriented, and this is an existential characteristic that makes human existence what it is. The term “hard-wired” is not a term that Heidegger would have used. I introduce it, however, to indicate the possibility of something going wrong in regard to being-with (see below). To the extent that the *Mitsein* structure is damaged, it damages the very core of the individual’s human existence.

The notion of an existential sociality is not only relevant to questions about social cognition and our relations to others. According to Husserl, the very *objectivity* of the world* as experienced* depends on others. This is what he refers to as transcendental intersubjectivity (e.g., Husserl, 1959, p. 449; Husserl, 1968, p. 295). Intersubjectivity is transcendental, in the sense that it is a condition of possibility for us to experience anything like a coherent and meaningful world, and specifically to experience it as real and objective. This latter point is what Husserl’s concept of transcendental intersubjectivity adds to Heidegger’s notion of *Mitsein*. The analysis Husserl gives is based on the perception of our immediate environment. We see things, not as mere surfaces, but as multi-sided objects based on an implicit reference to the (real or potential) perceptual perspectives that others can take on the same objects. Our basic experience of the world as having reality or objectivity depends on a kind of tacit confirmation by others.

Thus everything objective that stands before me in experience and primarily in perception has an apperceptive horizon of possible experience, including my own and that of others. Ontologically speaking, my perception of the world is, from the very beginning, part of an open but not explicit totality of possible perceptions [that others may also have]. The subjectivity belonging to this experience of the world is open intersubjectivity. (Husserl, 1973, p. 289; translated in Gallagher and Zahavi, 2012)

The idea that something may go wrong with the basic structures of being-with or transcendental intersubjectivity was followed up in the tradition of phenomenological psychiatry, as found in the classic works of Jaspers (1997), Minkowski (1970), Blankenburg (1971), and others. A certain form of derealization, for example, can be analyzed as a disruption of transcendental intersubjectivity, to the point that real things may no longer feel real or familiar, or as fully objective as they should. A significant privation of intersubjectivity, accordingly, may lead to an erosion of the sense of reality (although, to be sure, not all forms of derealization are due to such privation). Such experiences, found in instances of schizophrenia, may also be closely tied to the phenomenon of depersonalization. Phenomenologists have analyzed some of the symptoms of schizophrenia (including autistic aspects of schizophrenia) as involving very basic disruptions in self-experience or ipseity (e.g., Sass and Parnas, 2003). “Such experiences may also involve dissociative features, in which one experiences a pathological, subjective detachment from the external world, an estrangement from one’s body and even from mental processes” (Varga, 2012, p. 103). On this view, the loss of a basic intersubjective dimension of existence can lead to the loss of the sense of realness, as well as disturbances in what some have called the minimal self (Gallagher, 2000; Zahavi, 2007).

In terms of our actual engagement with others, being-with and transcendental intersubjectivity are cashed out in very basic sensory-motor processes involved in our bodily interactions with others. Merleau-Ponty calls this “intercorporeity”: “between this phenomenal body of mine and that of another … there exists an internal relation which causes the other to appear as the completion of the system” (Merleau-Ponty, 1962, p. 352). For Merleau-Ponty, our perception of others is interactional rather than observational, and the actions of others elicit the activation of our own motor systems. These processes involve the kind of motor resonance often described in the mirror neuron literature; but Merleau-Ponty emphasizes the dynamic interchange that one finds in the affective attunement that occurs between interacting agents. Merleau-Ponty’s notion of intercorporeity has been a special motivation for the more recently developed embodied and enactive approaches to perception and intersubjectivity found in the cognitive sciences (Varela et al., 1991; Noë, 2004). In this regard, Merleau-Ponty suggests that the borders of the transcendental and the empirical become indistinct – we should not think of the facticity of embodiment as external to subjective experience or cognition, but the place where the mind happens, or as he dramatically puts it, where “the transcendental descends into history” (Merleau-Ponty, 1967, p. 107). The best way to see the details of this kind of embodied intersubjectivity is by looking at developmental studies.

## DEVELOPMENTAL STUDIES

The *interaction theory* of social cognition draws on the work of the phenomenologists, but also the developmental studies of primary and secondary intersubjectivity (Trevarthen, 1979; also see Rochat, 2001; Hobson, 2004; Reddy, 2008). Primary intersubjectivity involves the sensory–motor capacities that shape our interactions with others from the very beginning. Just after birth, for example, infants are capable of interacting with others, as evidenced in experiments on neonatal imitation (Meltzoff and Moore, 1977).

Throughout the first year of life, infants develop an enactive perceptual access to the emotional and intentional states of others. At 2 months, for example, second-person *interaction* with others is evidenced by the timing of their movements and emotional responses. Infants “vocalize and gesture in a way that seems [affectively and temporally] “tuned” to the vocalizations and gestures of the other person” (Gopnik and Meltzoff, 1997, 131). Further evidence for this is provided by still face experiments (Tronick et al., 1978) and contingency studies (Murray and Trevarthen, 1985) where infants become significantly upset when faced with unresponsive behavior or mis-timed responses from the mother.

The concept of secondary intersubjectivity (Trevarthen and Hubley, 1978) is associated with the advent of joint attention during the first year. Infants start to notice how others pragmatically engage with the world and they begin to co-constitute the meaning of the world through interactions with others in joint actions. Pragmatic and social contexts start to matter and they enter into situations of participatory sense-making (De Jaegher and Di Paolo, 2007).

The important point made by interaction theory is that both primary and secondary intersubjectivity are not only early developing, characterizing our existence from infancy, but they remain essential aspects of our continued adult existence with others. Moreover, in processes of primary intersubjectivity we develop and continue to sustain a relational sense of self. That is, a sense of self that is intricately coupled to others. Neisser (1988) called this the interpersonal aspect of the self. If one’s primary, most basic, minimal sense of self is tied to one’s embodied, sensory-motor, proprioceptive processes, these processes are fully involved in intersubjective interactions from the start. We are, as Guenther (2013) puts it, *relationally constituted*.

All of these intersubjective dimensions are reflected later in the way we start to form our self-narratives, and our own narrative self. In contexts where infants are already interacting with their caregivers in personal and pragmatic relations, beginning narratives are elicited from 2-year-olds by questions and prompts (Howe, 2000), and “the child’s own experience … is forecast and rehearsed with him or her by parents …. [C]hildren of 2–4 years often “appropriate” someone else’s story as their own” (Nelson, 2003, p. 31). These developmental facts suggest the importance of the role played by narratives in our understanding of self and others – and they continue to be important throughout our adult life.

Again, it’s important to note that the capacities of primary and secondary intersubjectivity are not precursors; they are not left behind, but continue to characterize our mature adult behavior – supplemented and transformed via communicative and narrative practices. Behavioral analyses of social interactions in joint actions and shared activities, in working together, in communicative practices, and so on, show that adult agents unconsciously coordinate their movements, gestures, and speech acts (Kendon, 1990; Issartel et al., 2007; Lindblom and Ziemke, 2008). In communicative practices we coordinate our perception–action sequences; our movements and gestures are coupled with changes in velocity, direction and intonation in the movements, gestures and utterances of the other speaker.

Furthermore, the social interaction which characterizes primary and secondary intersubjectivity goes beyond each participant; it results in something (the creation of meaning) that goes beyond what each individual qua individual can bring to the process (De Jaegher et al., 2010). One can think of dance or the tango as a metaphor for the kind of dynamic production of meaning involved in interaction. In the tango something dynamic is created that neither individual could create alone. These interactive practices shape who we are; our identities; our meaningful experiences of the world; and what we take to be valuable or not so valuable.

## WHAT HAPPENS WHEN SUBJECTS ARE DEPRIVED OF INTERSUBJECTIVE INTERACTIONS?

### INDUCED AUTISM

During the Ceauşescu regime in Romania young children were left in orphanages, often because of the extreme poverty of their parents. Hobson (2004) summarizes the conditions in these orphanages:

• Infants were confined to their cots, without toys or other playthings, fed by bottles that were sometimes left propped up for use.
• Little sustained, interpersonal exchange; no opportunities to establish relationships with caregivers.
• The physical environment was also extremely harsh, and it was not uncommon for children to be washed by being hosed down with cold water.

Children from these orphanages tended to lack the reciprocal to and fro of social exchange, they showed limited social awareness and empathy, they found it difficult to maintain social interaction, and they would rarely turn to their adoptive parents for security and comfort (Hobson, 2004).

Studies by Rutter et al. (1999) showed that a small but much higher than expected proportion of these children developed an atypical (or quasi) form of early childhood autism. A variety of studies found severe problems with social relationships and communication involving

• Poverty of eye-to-eye gaze and gestures in social exchanges
• Limited language and to-and-fro conversation
• Preoccupations with sensations and other cognitive characteristics of autism – including strong interest in abnormal patterns and objects (problems in perception of Gestalt).

Additional studies show

• Emotional difficulties sustained through childhood (Colvert et al., 2008).
• Negative effects on motor development in children who were psychosocially deprived in these orphanages (Levin et al., 2014)
• Disruptions in the development of the neural circuitry involved in the recognition of facial expressions, due to psychosocial deprivation (Parker and Nelson, 2005)

Rutter et al. (1999) drew the tentative conclusion that prolonged experience of such terrible social and non-social privation was responsible for these quasi-autistic symptoms. Hobson is less tentative: the circumstances of these institutions led to a form of induced autism. Autism (naturally occurring or induced) involves “a disorder of the system of child-in-relation-to other” (Hobson, 2004, p. 203).

By looking at studies of naturally occurring autism we can be more specific about the embodied aspects involved in generating social deficits. There is extensive evidence to suggest that autism involves problems with basic sensory–motor processes that support primary intersubjectivity. Long-standing research based on the analysis of videos of infants younger than 1 year and later diagnosed with autism shows asymmetries or unusual sequencing in crawling and walking, as well as problems and delayed development in lying, righting, sitting (Teitelbaum et al., 1998). Recent studies by Elizabeth Torres et al. (2013) show in great detail disrupted patterns in re-entrant (afferent, proprioceptive) sensory feedback that usually contribute to the autonomous regulation and coordination of motor output. From an early age, this feedback supports volitional control and fluid, flexible transitions between intentional and spontaneous behaviors.

Torres shows that across the entire autistic spectrum there is a disruption in the maturation of this form of proprioception, accompanied by behavioral variability in motor control. In clear contrast to typically developing individuals, the normalized peak (micro-movement) velocity and noise-to-signal ratios of all participants with ASD, including adolescents (14–16 years old) and young adults (18–25 years old), across different ages and across verbal or non-verbal status remained in the region corresponding to younger typically developing children. In the motor system, noise overpowers signal in ASD. Proprioceptive input was random (unpredictable), noisy (unreliable), and non-diversified, and autistic subjects had difficulty distinguishing goal-directed from goal-less motions in most tasks (Torres et al., 2013, p. 16).

Accordingly, because proprioception is random, noisy, and restricted, it’s unlikely that individuals with ASD can anticipate the consequences of their own impending movements in a timely fashion. It’s also unlikely they could apply fine-tuned discriminations to the actions and emotional facial micro-expressions of others during real time social interactions – entailing a disruption of intercorporeity.

To be clear, my appeal to the data on motor problems in ASD is not meant to suggest an equivalency between individuals with ASD and those that have a form of induced or quasi-autism. Rather, the point is simply that some of the same motor difficulties that correlate with problems in social or intersubjective experience can be found in both groups. Furthermore, both of these groups are, or come to be, embedded in socially rich environments, and this clearly differentiates them from prisoners in solitary confinement who may develop similar motor problems (see below). Indeed, we’ll see that prisoners in solitary confinement are moving on the opposite trajectory: deprived of intersubjective contact, they sometimes develop very basic motor problems. In contrast, children who show signs of quasi-autism often improve once they are introduced into social and caring environments; likewise, some individuals with ASD who engage in social interactions improve their social performance and achieve a high level of intersubjective activity. The important point, in the context of this paper, is that motor problems that can undermine social interaction can be induced by social and physical privation.

### SOLITARY CONFINEMENT

Prisoners who are subjected to solitary confinement show symptoms and describe a phenomenology that is not equivalent to either autism or induced autism, but reflect similar motor problems, and often times more extensive and serious disruptions of experience. Guenther (2013), looking at the phenomenology associated with solitary confinement, describes it as becoming “unhinged”: “[Prisoners subjected to solitary confinement] see things that do not exist, and they fail to see things that do. Their sense of their own bodies – even the fundamental capacity to feel pain and to distinguish their own pain from that of others – erodes to the point where they are no longer sure if they are being harmed or are harming themselves” (2013, p. xi). There is a long list of experiences associated with solitary confinement: anxiety, fatigue, confusion, paranoia, depression, hallucinations, headaches, insomnia, trembling, apathy, stomach and muscle pains, oversensitivity to stimuli, feelings of inadequacy, inferiority, withdrawal, isolation, rage, anger, and aggression, difficulty in concentrating, dizziness, distortion of the sense of time, severe boredom, and impaired memory (Smith, 2006).

Peter Smith notes: “whether and how isolation damages people depends on duration and circumstances and is mediated by prisoners’ individual characteristics; but for many prisoners, the adverse effects are substantial” (2006, p. 441). He documents high rates of mental illness resulting from solitary confinement, starting in the nineteenth century. Hearing about new practices of solitary confinement in American prisons, delegates from Europe came to learn about it. One visitor, the author Charles Dickens, refers to solitary confinement as “slow and daily tampering with the mysteries of the brain … immeasurably worse than any torture of the body” (1957, 99; cited in Guenther, 2013, p. 18).

Studies of 100 inmates in California’s Pelican Bay Supermax prison (Haney, 2003) found 91% of the prisoners suffering from anxiety and nervousness; 70% “felt themselves on the verge of an emotional breakdown” (p. 133); 77% experience chronic depression. One prisoner reported the following experience:

I went to a standstill psychologically once – lapse of memory. I didn’t talk for 15 days. I couldn’t hear clearly. You can’t see – you’re blind – block out everything – disoriented, awareness is very bad (Cited in Grassian, 1983, 1453).

Another confirmed sensory disturbances:

Melting: Everything in the cell starts moving; everything gets darker, you feel you are losing your vision (Grassian, 1983, 1452).

And another confirmed memory problems.

Memory is going. You feel you are losing something you might not get back.” (Grassian, 1983, 1453).

A systematic review of the phenomenology of solitary confinement reveals symptoms that involve serious bodily and motor problems, derealization, and self-dissolution (or depersonalization).

#### Bodily and motor problems

Dickens, when visiting an American prison, was curious about the trembling of the prisoners in solitary confinement – “their nervous ticks, their difficulty in meeting his eye or sustaining conversation, their cringing posture and nervousness …” (Guenther, 2013, p. 19). To Dickens’ observation a prison guard replied:

Well it’s not so much a trembling, although they do quiver – as a complete derangement of the nervous system. They can’t sign their names to the book; sometimes they can’t even hold the pen … sometimes they get up and down again twenty times in a minute…. Sometimes they stagger as if they were drunk, and sometimes are forced to lean against the fence, they’re so bad (Dickens, 1957, pp. 105–106).

Dickens adds that the prisoner’s sensory awareness, their capacities to see and hear clearly, to make sense of their perceptions were diminished. “That it makes the senses dull, and by degrees impairs the bodily faculties, I am quite sure” (Dickens, 1957, pp. 108–109). Guenther is right that “it is precisely at the level of bodily perception, sensibility and affectivity that prisoners find their relation to the world undermined” (2013, p. 154).

It’s a question open to future empirical investigation whether this kind of undermining of embodiment is similar to the sensory–motor problems described by Torres (2013) in terms of disrupted patterns in the peripheral nervous system – disruptions of the re-entrant (afferent, proprioceptive) sensory feedback that usually contribute to the autonomous regulation and coordination of motor output, as well as to primary intersubjectivity. The observed symptoms do seem similar: poverty of eye-to-eye gaze and gestures in social exchanges; limited language and to-and-fro conversation; a variety of sensory-motor problems.

#### Derealization

One also finds, correlatively, reports from prisoners in solitary confinement reflecting a derealization – undermining their relation to the world. Thus, the experience of object boundaries becomes uncertain.

It becomes difficult to tell what is real and what is only my imagination playing tricks on me…. the wire mesh on [the] door begins to vibrate or the surface of the wall seems to bulge. (Guenther, 2013, p. 35; citing Grassian, 1983; Shalev, 2009).

As Guenther suggests, in solitary confinement the transcendental intersubjective basis of the experience of the world as real and objective is structurally undermined (2013, p. 35). It completely closes down the possibility of secondary intersubjectivity and therefore of participatory sense making, undermining the capacity to sustain meaning. These problems with derealization, and with sensory-motor processes, correlate with depersonalization and the dissolution of the self.

#### Self-dissolution

Christensen, in a study of a woman who experienced solitary confinement in Denmark, writes: “The person subjected to solitary confinement risks losing her self and disappearing into a non-existence” (Christensen, 1999, p. 45; cited and trans. by Smith, 2006, p. 497). It is important, however, to specify precisely what aspects of self are at stake in such a statement. Guenther (2013, p. xiii) gives a better indication when she asks: “How could I lose myself by being confined to myself? For this to be possible, there must be more to selfhood than individuality …. Solitary confinement works by turning prisoners’ constitutive relationality against themselves.” That is, solitary confinement disrupts the *relational self* by disrupting primary and secondary intersubjectivity, and the intercorporeity essential to social interaction.

The practice of solitary confinement is not, as some of the original prison administrators thought, a way for the prisoner to return into self – “The inmate was expected to turn his thoughts inward …”– a rehabilitation through isolation with oneself (Smith, 2006, p. 456; see Guenther, 2013, p. xvi). Such a proposal reflects a traditional concept of self as an isolated individual substance or soul that benefits from introspection. If, in contrast, the self is relational, then solitary confinement, by undermining intersubjective relationality, leads to a destruction of the self. Stripping away the possibility of primary intersubjectivity – leading to the experience of depersonalization – goes to the very basic level of the *minimal embodied self*.

It also affects the *narrative self*. Self-narrative depends on having something to narrate, and having someone to whom to narrate. In addition, self-narrative practices require four distinct capacities (Gallagher, 2007):

(1) The capacity for temporal ordering. This involves two aspects of temporality. The ability to order events serially (within the narrative) – a temporality associated with what McTaggart (1908) called the “B-series” of earlier-than and later-than; and the ability to maintain a temporal perspective on one’s own narrating activities – a temporality associated with the A-series of constantly changing relations between past, present, and future. The self who narrates about past things from a present perspective (A-series), for example, needs to be able to enact a serial order in the narrated events (B-series).
(2) The capacity for minimal self-reference. To begin to form a self-narrative one must be able to refer to oneself by using the first-person pronoun. Without the basic (and basically embodied and agentive) sense of differentiation between self and non-self I would not be able to refer to myself with any specification, and self-narrative would have no starting point. The minimal sense of self, closely tied to embodied existence, is what gets extended and enhanced in the self-narrative.
(3) Episodic and autobiographical memory. Both the capacity for temporal ordering and the capacity for minimal self-reference are necessary for the proper working of episodic and autobiographical memory, which involves the recollection of a past event and when it took place, and self-attribution, the specification that the past event involves the person who is remembering it. Whatever degree of unity my life has, it is the product of an interpretation of my past actions and of events in the past that happened to me, all of which constitute my life history (Ricoeur, 1992). If I am unable to form or access memories of my life history, then I have nothing to interpret, nothing to narrate that would be sufficient for the continuity of self-identity.
(4) The capacity for metacognition, that is, an ability to gain a reflective distance from one’s own experience. The process of interpretation that ordinarily shapes episodic memories into a narrative structure depends on this capacity. To form a self-narrative, one needs to reflectively consider one’s life events, deliberate on their meaning, and decide how they fit together semantically. A life event is not meaningful in itself; rather it depends on a narrative structure that lends it context and sees in it significance that goes beyond the event itself. As Donald (2006) puts it, metacognition provides the “cognitive governance” that allows for disambiguating and differentiating events within the narrative.

As it turns out, all four capacities are under threat in the context of solitary confinement. Among the commonly reported symptoms that result from solitary confinement are distortions in the sense of time, which can clearly affect the capacity for temporal ordering; basic disruptions in bodily integrity, so that differentiation between self and non-self is compromised (Guenther, 2013, p. xi); impaired memory; and cognitive difficulties (concentration, confusion) that will clearly affect metacognition.

One can understand the self as a pattern of various aspects (Gallagher, 2013), some of which we have named as minimal embodied aspects, relational aspects, and narratival aspects. On the pattern theory of self, what we call self consists of a complex pattern of a sufficient number of contributories, none of which on their own is necessary or essential to any particular self. Taken together, a certain pattern of characteristic features constitutes an individual self. Such patterns may change over time, taking on different weights and values for the individual they define, and for others, who normally have an influence on how the pattern unfolds. The pattern includes minimal embodied and experiential aspects, affective aspects, intersubjective or relational aspects, psychological/cognitive aspects, narrative aspects, extended aspects, and situational aspects (Gallagher, 2013). Extended aspects include those things that an individual has invested in or considers his own, as James (1890, p. 279) suggested: “*a man’s Self is the sum total of all that he* CAN* call his*, not only his body and his psychic powers, but his clothes and his house, his wife and children, his ancestors and friends, his reputation and works [etc.]”. Situational aspects include aspects that play some (major or minor) role in shaping who we are, including the kind of family structure and environment where we grew up; cultural and normative practices that define our way of living, but even the physical surroundings that offer affordances or disaffordances for action.

The evidence reviewed above suggests that solitary confinement negatively affects all of these aspects. Reports from prisoners, medical personnel, psychologists, and psychiatrists suggest serious problems with minimal embodied aspects (e.g., physical health and motor problems), experiential aspects (e.g., sensory problems, derealization), affective aspects (e.g., depression, anxiety), intersubjective or relational aspects (e.g., isolation), psychological/cognitive aspects (e.g., lack of concentration, confusion), narrative aspects (e.g., memory problems, distortions in time sense), extended aspects (e.g., lack of control over personal property), and situational aspects (e.g., relatively dire circumstances in prison cells). A breakdown in some significant number of these aspects would be sufficient to alter, or even eradicate the pattern that constitutes self in any particular case.

## CLARIFYING THE NOTIONS OF CRUEL AND UNUSUAL

The words “cruel and unusual punishment” first appeared in the English Bill of Rights in 1689. As initially noted, they also appear in the Eighth Amendment to the United States Constitution (1791): “cruel and unusual punishments [shall not be] inflicted.” On the British side, the term “cruel” was synonymous with “severe,” and generally signified punishments that were disproportionate to the crime (Granucci, 1969, p. 860). The American interpretation, in contrast, focused on identifying cruel methods (and specifically torturous methods) of punishment (see e.g., Berkson, 1975)^3^. Unfortunately, some cruel and unusual punishment is not so unusual – so we may prefer the wording of the Universal Declaration of Human Rights adopted by the UN General Assembly (A/RES/217, 1948): “No one shall be subjected to torture or to cruel, inhuman or degrading treatment or punishment.” This still leaves us with the question of what constitutes cruel, inhuman or degrading punishment, and as noted at the start it is still difficult to find a clear definition of these terms in the legal domain.

In 1972, United States Supreme Court Justice William Brennan, in *Furman v. Georgia* (408 U.S. 238; 1972), a case involving the death penalty, defined four principles that determine when a punishment is cruel and unusual:

1. When the severity is ***degrading*** to human dignity (including torture).
2. When a severe punishment is “obviously inflicted in wholly ***arbitrary*** fashion.”
3. When the severe punishment “is clearly and totally ***rejected throughout society***.”
4. When a severe punishment is “patently ***unnecessary***.”

Unlike the first principle, principles 2, 3, and 4 are easier to measure or define. It’s not clear, however, that on their own, arbitrariness, social rejection, and lack of necessity define the concept of cruelty. Justice Brennan thus suggests that these principles need to be applied in a convergent fashion. That the concept of “cruelty” (or “degrading to human dignity”^4^) remains obscure can be seen in how it is glossed in the following explanation.

[These criteria are] interrelated, and, in most cases, it will be their convergence that will justify the conclusion that a punishment is “cruel and unusual.” The test, then, will ordinarily be a cumulative one: if a punishment is *unusually severe*, if there is a strong probability that it is inflicted arbitrarily, if it is substantially rejected by contemporary society, and if there is no reason to believe that it serves any penal purpose more effectively than some less severe punishment, then the continued infliction of that punishment violates the command of the Clause that the State may not inflict inhuman and uncivilized punishments upon those convicted of crimes. (Brennan, 1972, p. 239; emphasis added)

The severity of punishment that is degrading to human dignity is explicated as when the severity is “unusually severe.”

Without dismissing the other three principles, I want to suggest that the phenomenology of solitary confinement provides a clearer interpretation of the concept of *cruelty* or *degrading of human dignity,* one that on its own should be sufficient for disqualifying solitary confinement as an acceptable punishment^5^.

The concept of self or person that the liberal tradition sets up as having dignity and demanding respect is a standard that treats the self as a stand-alone individual capable of autonomous deliberation and decision (see e.g., Code, 2011). Both phenomenology and science shows this to be an abstraction that fails to recognize the *relational* nature of the self with embodied, experiential, and affective dimensions, complicated by narrative, extended and situated aspects of human existence. Solitary confinement morally degrades human dignity by literally degrading (if not destroying) the human self in all of these aspects, starting with the deeply relational dimension. Ethically and practically speaking, this multi-dimensional, relational self is the only viable concept of self that the liberal tradition should use to measure its own practices pertaining to dignity, respect, and justice. If we destroy the self in its full pattern, or in a sufficient number of its aspects, it would be difficult to argue that we are respecting the person in any moral sense and not degrading the dignity of the human being.

## Conflict of Interest Statement

The author declares that the research was conducted in the absence of any commercial or financial relationships that could be construed as a potential conflict of interest.

## Footnotes

1 Granucci, (1969, p. 842); citing representatives to the First Congress, Smith and Livermore, respectively. Granucci provides a fine-grained history of the phrase.

2 This is clearly a different hermeneutical procedure than found in most legal considerations where appeal is often to historical meaning or to evolving moral standards on such questions.

3 In Coker vs Georgia (433 U.S. 584 [1977]), however, the US Supreme Court interpreted the phrase in terms of disproportionality (Radin, 1978). Most legal interpretations of this phrase are tied to the death penalty. The range of interpretation of what constitutes “cruel and unusual” is wide, however. Thus, MacReady (2009, 708) reports, in *The Lancet*, “Substandard prison health care is deemed a violation of the Eighth Amendment to the Constitution that prohibits cruel and unusual punishment, making prisoners the only group of Americans who are guaranteed medical care.” For more on the history and background on the legal issues concerning cruel and unusual punishment and solitary confinement, see Dayan and Dayan (2007); Madrid v Gomez (889 F. Supp. 1146 – Dist. Court, ND California, 1995), a case in which a district court judge came close to condemning solitary confinement as cruel and unusual; also Wedekind, 2011 and Solitary Watch (n.d.).

4 The concept of dignity is not well defined in the law either (see McDougal et al., 1980). Pellegrino (2008, p. xi) states: “… there is no universal agreement on the meaning of the term, human dignity.” The term in used in a variety of ways, but it is often associated with the concept of respect for the human person.

5 There is no universal agreement that solitary confinement need be considered cruel and unusual punishment. Thus, Bonta and Gendreau (1990), who discount phenomenological and qualitative studies in favor of more objective and experimental ones, conclude: “solitary confinement may not be cruel and unusual punishment under the humane and time-limited conditions investigated in experimental studies or in correctional jurisdictions that have well-defined and effectively administered ethical guidelines for its use” (p. 361). Bonta and Gendreau’s study, however, has been subject to widespread criticism [see, for example, the critique by Jackson (2002)]. Furthermore, most psychiatric studies of solitary confinement have condemned the practice (Grassian, 1983; Haney, 2003). Note that objective research of prison conditions, including solitary confinement, is very difficult to undertake, given security constraints. Most studies of solitary confinement are commissioned as parts of lawsuits (e.g., Grassian and Haney have both testified as expert witnesses).
